# The Complexity of SARS-CoV-2 Infection and the COVID-19 Pandemic

**DOI:** 10.3389/fmicb.2022.789882

**Published:** 2022-02-10

**Authors:** Maria Karoliny da Silva Torres, Carlos David Araújo Bichara, Maria de Nazaré do Socorro de Almeida, Mariana Cayres Vallinoto, Maria Alice Freitas Queiroz, Izaura Maria Vieira Cayres Vallinoto, Eduardo José Melo dos Santos, Carlos Alberto Marques de Carvalho, Antonio Carlos R. Vallinoto

**Affiliations:** ^1^Laboratory of Virology, Institute of Biological Sciences, Federal University of Pará, Belém, Brazil; ^2^Graduate Program in Biology of Infectious and Parasitic Agents, Federal University of Pará, Belém, Brazil; ^3^Laboratory of Complex Diseases, Institute of Biological Sciences, Federal University of Pará, Belém, Brazil; ^4^University Center of the State of Pará, Belém, Brazil; ^5^Center for Biological and Health Sciences, Department of Pathology, State University of Pará, Belém, Brazil

**Keywords:** SARS-CoV-2, COVID-19, coronavirus, pandemic, infection

## Abstract

The pandemic caused by the severe acute respiratory syndrome coronavirus 2 (SARS-CoV-2) led to the death of millions of people worldwide and thousands more infected individuals developed sequelae due to the disease of the new coronavirus of 2019 (COVID-19). The development of several studies has contributed to the knowledge about the evolution of SARS-CoV2 infection and the disease to more severe forms. Despite this information being debated in the scientific literature, many mechanisms still need to be better understood in order to control the spread of the virus and treat clinical cases of COVID-19. In this article, we carried out an extensive literature review in order to bring together, in a single article, the biological, social, genetic, diagnostic, therapeutic, immunization, and even socioeconomic aspects that impact the SAR-CoV-2 pandemic. This information gathered in this article will enable a broad and consistent reading of the main aspects related to the current pandemic.

## Introduction

On December 8, 2019, a viral infection, previously unidentified, characterized by severe pneumonia, was reported in an individual who frequented a small local fish and wildlife market in the city of Wuhan, Hubei Province, China (Lu H. et al., 2020). The analysis based on nucleotide sequencing technology of the virus genome isolated from the blood of sick individuals led to the identification of a new coronavirus as the causative agent of the outbreak (Gorbalenya et al., 2020). Initially, the infected individuals were those who visited the seafood market or consumed foods of animal origin probably infected with severe acute respiratory syndrome coronavirus 2 (SARS-CoV-2). Later, a more in-depth analysis through contact tracing of patients positive for COVID-19 revealed that several individuals with no history of trips to the seafood market also tested positive for the disease, indicating the possibility of transmission from person to person (Chan J. F. et al., 2020; Chen J. et al., 2020; Ji et al., 2020).

During the second week of January 2020, due to the travel season of the Spring Festival, the new SARS-CoV-2 spread to other provinces of China and thus to other countries (Lu H. et al., 2020). The first case of SARS-CoV-2 infection confirmed outside of China was in Thailand on January 13, 2020. On January 16, 2020, the first case was confirmed in Japan. As of January 25, 2020, the number of confirmed cases had reached 2062, including in countries such as Hong Kong, Macao, Australia, Malaysia, Singapore, France, South Korea, Taiwan, United States, Vietnam, Nepal, and Sweden (Dhama et al., 2020). Due to the severity of this outbreak and its ability to spread internationally, the World Health Organization (WHO) declared a global health emergency on January 31, 2020. On March 11, 2020, a pandemic was declared (Cucinotta and Vanelli, 2020). Data published by the WHO showed that up to December 3, 2021, 22,105,872 people had been confirmed infected by the new coronavirus, 614,964 had died, and 312,827,402 doses of vaccine were administered (World Health Organization [WHO], 2021a).

This review aims to explore and summarize the available evidence on the main viral characteristics, immune response, diagnostic methods, therapeutic options and candidate/approved vaccines against SARS-CoV-2, so that this information can serve as a basis for a better understanding of future studies on SARS-CoV-2 and COVID-19.

## SARS-CoV-2

### Morphological, Genomic Structure and Replication of SARS-CoV-2

**FIGURE 1:**
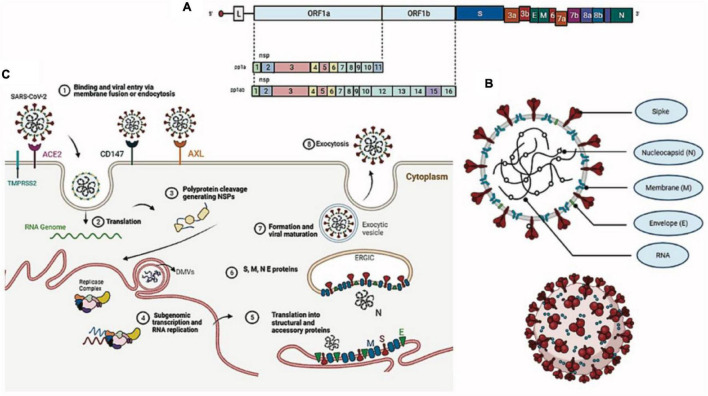
Morphological structure, genome, and replication of SARS-CoV-2. **(A)** Viral genome. **(B)** Viral particle. **(C)** SARS-CoV-2 replication cycle.

Coronaviruses are spherical, enveloped viruses of approximately 120 nm in diameter, containing a helical symmetry nucleocapsid, with a single-stranded RNA genome of positive polarity, non-segmented, 29.9 kb in size (NC_045512.2), and a GC content of 38% (Chan K. et al., 2020; Figure 1B). Its genome is composed of 13 open reading frames (ORFs) (Lu R. et al., 2020) encoding 7096 amino acids that constitute four structural proteins spike (S), envelope (E), membrane (M), and nucleocapsid (N) (Table 1) and 15 non-structural proteins (NSP1, NSP2, NSP3, NSP4, NSP5, NSP6, NSP7, NSP8, NSP9, NSP10, NSP12, NSP13, NSP14, NSP15, and NSP16), in addition to eight accessory proteins (3a, 3b, 6, 7a, 7b, 8b, 9b, and ORF14) that perform numerous functions in the processes of virus replication and assembly (Wu A. et al., 2020; Figure 1A). In comparison to SARS-CoV, SARS-CoV-2 lacks protein 8a but has a longer 8b protein, with approximately 121 amino acids, and a shorter 3b protein, containing 22 amino acids (Chan K. et al., 2020; Wu A. et al., 2020). More than 380 amino acid substitutions, located mainly in the NSP3, NSP2, and S proteins, have been identified between SARS-CoV-2 isolates and the consensus sequence, leading to divergence in the functional and pathogenic traits between it and other coronaviruses (Wu A. et al., 2020). We synthesized the role of structural proteins in the SARS-CoV-2 replication cycle (Table 1).

**TABLE 1:** SARS-CoV-2 structural proteins and their roles in the viral replication cycle.

Protein	Function	References
Spike (S)	Divided into two subunits (S1 and S2) it is able to bind to the host cell through its receptor-binding domain. The S1 subunit is involved in binding the virus to the host cell membrane, while the S2 subunit acts in fusing the viral envelope with the cell membrane.	Hoffmann et al., 2020; Lan et al., 2020; Wrapp et al., 2020
Nucleocapsid (N)	It binds and packages viral RNA into ribonucleoprotein complexes (RNP) located inside the viral envelope, forming a separate layer from the M, E, and S envelope proteins. This protein is recruited into the replication-transcription complex by NSP3 and, therefore, it is believed to be also involved in viral genome replication.	Chang et al., 2020; Cong et al., 2020; Yao et al., 2020
Envelope (E)	Protein E has a transmembrane domain and is relatively small in size, with about 75 amino acids, which aid in the assembly and release of virions.	Nieto-Torres et al., 2015; Venkatagopalan et al., 2015; Lu R. et al., 2020
Membrane (M)	It has about 222 amino acids and is the most present protein in the viral particle, giving definitive shape to the virion envelope. This protein works simultaneously with proteins E, N, and S and plays an important role in ribonucleic acid (RNA) packaging and virus assembly.	Tang X. et al., 2020

The SARS-CoV-2 replication cycle begins with the binding of the S protein of the virus with the angiotensin-2 converting enzyme (ACE-2) of the host, considered the specific receptor of SARS-CoV-2 in human cells (Hoffmann et al., 2020). ACE2 is a type I membrane glycoprotein responsible for the conversion of angiotensin II into angiotensin 1–7 and is expressed in the lungs, nose, heart, intestine, and kidneys (Yan et al., 2020). The S protein of SARS-CoV-2 is a class I fusion protein that protects its fusion domain, keeping it hidden and inactive until the virus finds a host cell with its receptor, where it is then proteolytically cleaved into a hook-shaped structure that is necessary for its incorporation into the membrane of the target cell (Li and Petrovsky, 2016). The binding of the S protein to ACE-2 is dominated by polar contacts mediated by hydrophilic residues located in its C-terminal domain and promotes a cleavage event dependent on the endosomal cysteine proteases CatB and CatL or the transmembrane serine protease TMPRSS2, which exposes the protein S fusion peptide in order to favor viral entry (Hoffmann et al., 2020; Wang et al., 2020a; Yan et al., 2020). Two possible new SARS-CoV-2 receptors were recently identified: the tyrosine kinase AXL receptor and the transmembrane surface glycoprotein CD147 (inducer of extracellular matrix metalloproteinase or basigin), both capable of interacting with the S protein (Wang et al., 2020b; Wang S. et al., 2021). In addition, the cell receptor neuropilin-1 (NRP1), expressed in the olfactory epithelium, also seems to facilitate SARS-CoV-2 infection by interacting with the S protein (Daly et al., 2020).

After viral entry, the SARS-CoV-2 genomic RNA serves as a transcript that allows the translation of two polyproteins (pp1a and pp1ab), encoded in the 3′ two-thirds of the genome as ORF1a and ORF1b (Zhou P. et al., 2020). These polyproteins are cleaved by the action of two viral proteases (NSP3-PLpro and NSP5-Mpro), generating 16 non-structural proteins that are assembled into the replicase–transcriptase complex, which will later give rise to genomic and subgenomic RNAs (Fehr and Perlman, 2015). NSPs also induce cell membrane rearrangement to form double-membrane vesicles (DMVs), where the replication-transcription complex (RTC) is anchored (Ziebuhr et al., 2000; Angelini et al., 2013). The other third of the genome, at the 5′ end, encodes structural (S, E, M, and N) and accessory proteins (3a, 3b, 6, 7a, 7b, 8b, 9b, and ORF14) (Siu et al., 2008; Fehr and Perlman, 2015; Wu A. et al., 2020). In addition to complexes between viral proteins, different interaction complexes are formed between structural and non-structural proteins of the virus and host cell proteins (Srinivasan et al., 2020).

The replication of SARS-CoV-2 is a complex process that involves atypical RNA revision by NSP14, one of the non-structural proteins generated by the cleavage of the pp1ab polyprotein (Romano et al., 2020). Such a revision mechanism present in coronaviruses, unknown among RNA viruses before their discovery in SARS-CoV, results in replication error rates more than 10 times lower than those of other RNA viruses (approximately 10^–6^ mutations/nucleotide/cycle/nucleotide/cycle) (Rausch et al., 2020).

Structural proteins translated from subgenomic mRNAs are inserted into the endoplasmic reticulum (ER) and pass through the secretory pathway to the ER–Golgi intermediate compartment (ERGIC), where the S protein is cleaved into two subunits, S1 and S2 (Jaimes et al., 2020). The newly synthesized viral genome forms a complex with the N protein in the ERGIC for the assembly of new SARS-CoV-2 particles in an event mediated by the M protein with contributions from the E protein (Siu et al., 2008; Tseng et al., 2013). New viral particles emerge from ERGIC and are transported through vesicles to be released into the extracellular medium by exocytosis (Ulasli et al., 2010; Figure 1C).

### Taxonomic Classification

Coronaviruses belong to the family *Coronaviridae*, which contains four genera (*Alphacoronavirus*, *Betacoronavirus*, *Deltacoronavirus*, and *Gammacoronavirus*) and includes species with a single-stranded RNA genome with positive polarity and 26–32 kb (International Committee on Taxonomy of Viruses [ICTV], 2019). SARS-CoV-2 belongs to the B strain of the genus *Betacoronavirus*, whose members infect only mammals (International Committee on Taxonomy of Viruses [ICTV], 2020; Mittal et al., 2020). Coronaviruses are zoonotic viruses with high mutation rates that infect a wide variety of wild and domestic animals and can also infect humans. Evolutionary analyses have shown that *Alphacoronavirus* and *Betacoronavirus* have bats and rodents as reservoirs, while birds are possible reservoirs of *Deltacoronavirus* and *Gammacoronavirus* (Chan et al., 2013, 2015).

### The Origin

Until 2002, coronaviruses were not known to cause serious infections in humans. This scenario changed with the emergence of SARS-CoV in an animal market located in southern China, which later affected more than 8,000 people, with 774 deaths worldwide (Drosten et al., 2003; Lau et al., 2003). In 2012, a new coronavirus was identified as responsible for Middle East respiratory syndrome (MERS-CoV), infecting more than 2428 individuals and killing 838 (Zaki et al., 2012). SARS-CoV and MERS-CoV originated from bats and then jumped to another mammalian host, the civet of Himalayan palms (*Paguma larvata*) in the case of SARS-CoV and the dromedary camel (*Camelus dromedarius*) in the case of MERS-CoV, before infecting humans (Song et al., 2005; Azhar et al., 2014).

SARS-CoV-2 is the third beta-coronavirus to infect humans. Identified in late 2019, it probably originated from bats and, over time, accumulated mutations that gave it the capacity for zoonotic transmission (Zhou P. et al., 2020). The bat coronavirus RaTG13 seems to be the closest relative to SARS-CoV-2, since it shares 93.1% identity in the nucleotide sequence of the S gene and 98% identity in the amino acid sequence of the S protein (Wrapp et al., 2020; Zhou P. et al., 2020). The transmission route of SARS-CoV-2 (or its direct ancestor) from bats to humans, either directly or through an intermediate animal species, remains undefined (Banerjee et al., 2021). The complete genomic sequences of SARS-CoV-2 obtained from five patients at an early stage of the outbreak were almost identical, and they had 79.6% similarity with the SARS-CoV sequences (Li et al., 2020; Xu et al., 2020). Initially, the virus was named new coronavirus 2019 (2019-nCoV). On February 11, 2020, the Coronavirus Study Group of the International Committee of Virus Taxonomy officially named it SARS-CoV-2 based on phylogenetic analyses that showed similarity with SARS-CoV (Gorbalenya et al., 2020).

### Genetic Variants of SARS-CoV-2

Since the beginning of the pandemic, the genome plasticity of SARS-CoV-2 was evidenciated with the detection of multiple sites in the genome under positive selection (Velazquez-Salinas et al., 2020). Viruses belonging to the same strain, but containing different subsets of mutations, can be classified as different variants that are characterized by their transmissibility, disease severity, and ability to escape humoral immunity. Increased transmissibility is demonstrated by the ability of a variant to compete with other variants and to exhibit a higher effective reproduction rate and/or secondary attack rate compared to other circulating variants (Korber et al., 2020; Volz et al., 2021). Next, we summarize the molecular characteristics of the main variants of SARS-CoV-2 (Table 2).

**TABLE 2:** Description/features of SARS-CoV-2 variants of concern.

Variant/WHO label	Lineage	Description	Additional aminoacid/key mutations
Alpha (Davies et al., 2021; Galloway et al., 2021; Volz et al., 2021; Walensky et al., 2021)	B. 1. 1. 7	UK lineage of concern, associated with the N501Y mutation United Kingdom, September-2020 +++ Transmissibility ++ Severity	+S: 484K +S:452R N501Y P681H Deletions: H69-V70 Y144/145
Beta (Tegally et al., 2021; Wibmer et al., 2021)	B. 1. 351	South Africa, May-2020 +Transmissibility Severity: possible	+S:L18F N501Y K417N E484K
Gamma (Candido et al., 2021; Wang P. et al., 2021)	P.1	Brazilian lineage with a number of spike mutations with likely functional significance E484K, K417T, and N501Y. Brazil, Dez-2020 ++ Transmissibility Severity: possible	+S:681H N501Y K417T E484K
Delta (European Centre for Disease Prevention and Control [ECDC], 2021; Motozono et al., 2021)	B. 1. 617. 2	Predominantly India lineage with several spike mutations. India, October-2020 +++ Transmissibility +++ Severity	+S:417N +S:484K L452R E484Q
Omicron (Callaway, 2021; Gao et al., 2021)	B. 1. 1. 529	Several mutations that are found in other variants of concern and that are thought to make the virus more infectious. Multiple countries, November-2021	D614G N501Y K417N

The mutations identified throughout the pandemic in the morphology of the virus clearly demonstrate, in addition to its adaptive capacity, its ability to develop evolutions in order to increase its ability to escape the host’s immune response, as well as make its entry into the cell easier, such mutations imply an increase in transmissibility or harmful alteration in the epidemiology of COVID-19; an increase in virulence or change in the clinical presentation of the disease; and/or diminished effectiveness of social and public health measures or available diagnostics, vaccines and therapies. Therefore, the wide vaccination coverage of the world population and the maintenance of measures to control the spread of the virus are the only efficient measures to contain these evolutions in the pathogenesis of the virus and in the effective control of the pandemic.

## Genetics of the Human Host in SARS-CoV-2 Infection

The role of human host genetic variability in the evolution of SARS-CoV-2 infection has been extensively proposed due to the great heterogeneity in the clinical manifestations of COVID-19 and the variation in mortality rates between populations and ethnicities, which are strong indicators of the modulatory effect of host genetics on its pathogenesis (Chakravarty, 2021; Fricke-Galindo and Falfán-Valencia, 2021; Mohammadpour et al., 2021).

Studies on the genetic predisposition to COVID-19 have been of various kinds, such as meta-analyses (Benetti et al., 2020; Poulton et al., 2020; Rendeiro et al., 2020; Bernal et al., 2021), *in silico* approaches (Vique-Sanchez, 2020; Wang et al., 2020c; Calcagnile et al., 2021), *in vitro* (Hashizume et al., 2021), case–control studies (Amraei et al., 2020; Sakuraba et al., 2020), and case series (Asselta et al., 2020; Russo et al., 2020; Wang et al., 2020d).

In case–control studies, there are two main approaches: (i) those rationally based on functional evidence (Lambert et al., 2005; Heurich et al., 2014; Cao et al., 2020; Maucourant et al., 2020; Wang et al., 2020d; Secolin et al., 2021), which search for specific candidate genes and investigate allele frequencies or differences in gene expression levels; and (ii) those based on genomic searches, such as whole-genome sequencing (WGS) (Wang et al., 2020f), whole-exome sequencing (WES) Zhang et al., 2020b; Kosmicki et al., 2021), and genome-wide association studies (GWAS) (Ellinghaus et al., 2020; Hu J. et al., 2020; Pairo-Castineira et al., 2021). Supplementary File organizes the associations reported in the literature by cytogenetic location, discriminating the evidence of association by type of study (WGS, WES, GWAS, case-control, meta-analyses, and functional evidence). A total of 51 regions could be identified containing approximately 86 candidate genes.

In this context, altered immune responses, such as those caused by primary immunodeficiencies (PIDs), may be important in disease progression, where at least one case of death has already been reported during coronavirus infection (Szczawinska-Poplonyk et al., 2013). More severe viral infections have been associated with the presence of PID (Dropulic and Cohen, 2011), which constitute a group of more than 350 rare diseases that together have a considerable prevalence (McCusker et al., 2018). Genetically, PIDs are heterogeneous, most often monogenic. The registry of rare diseases ORPHANET^1^ recognizes at least 308 genes involved in PIDs, which makes their diagnosis complex and their prevalence underestimated.

Total exome sequencing of SARS-CoV-2 identified 24 functional variants in eight genes, TLR3, UNC93B1, TICAM1, TBK1, IRF7, IFNAR2, IRF3, and IFNAR1 (Zhang et al., 2020a), of which the first six are on the list of 308 genes known to be associated with PID. Additionally, the ADAM17 gene, which is also on the list, has been implicated in the ability of SARS-CoV-2 to infect cells and modulate the inflammatory response (Heurich et al., 2014; Palau et al., 2020; Zipeto et al., 2020).

Few studies have addressed the direct relationship between PID and COVID-19 directly. A meta-analysis suggests a correlation between the prevalence of selective IgA deficiency, the most common PID, and the prevalence of COVID-19 (Naito et al., 2020). A case series with two men from two families indicated that mutations in the *TLR7* gene, one of those implicated in PID, were present in these patients with severe COVID-19 (Van der Made et al., 2020). An important prospective study suggests a 10-fold higher mortality rate from COVID-19 in children with PID (Delavari et al., 2020).

Together, these observations show that studies on the genetic modulation of COVID-19 by PID-related genes are consistent. Thus, Supplementary File shows that of the 51 chromosomal regions associated with COVID-19, 37 (72.5%) also contain PID-related genes, as described in ORPHANET. Among these 51 regions, those present on chromosomes 6, 19, and 21 stand out. On chromosome 6, more specifically in the MHC class I and II regions (6p21.32, 6p21.33, 6p22.1), the evidence of association with COVID-19 comes from WGS, GWAS, functional (expression and affinity) studies, meta-analyses, and case–control studies, and this region contains several PID-related genes. On chromosome 19, the most consistently associated region is the one containing the KIR gene complex (19q13.42), whose evidence comes from case–control, meta-analysis, and functional studies (Sakuraba et al., 2020; Bernal et al., 2021; Rendeiro et al., 2021) and which contains PID-related genes. Two regions of chromosome 21 (21q22.11, 21q22.3) also have multiple lines of evidence showing an association with COVID-19, the most strongly associated being the one that contains the *TMPRSS2* and *MX1* genes. *TMPRSS2* is already well established as an important marker for the ease of viral entry (Andolfo et al., 2020; Asselta et al., 2020; Latini et al., 2020; Sajuthi et al., 2020; Senapati et al., 2020; Vastrad et al., 2020; Wang et al., 2020c; Mohammad et al., 2021; Schönfelder et al., 2021; Torre-Fuentes et al., 2021), while *MX1* is a gene responsive to interferon and is closely directed to the activity of response to viral infections, including SARS-CoV-2 infection (Russo et al., 2020; Bizzotto et al., 2020).

The regulation of important genes in COVID-19 has also been addressed by epigenetic studies, which have shown that methylation patterns (Corley and Ndhlovu, 2020) and microRNA expression (Fulzele et al., 2020; Widiasta et al., 2020) are altered in the disease. The roles of microRNAs in the regulation of key genes in COVID-19 make them important candidate biomarkers. Another important aspect of their action is their binding to viral mRNAs to silence them and act as antivirals, as shown by *in silico* studies (Fulzele et al., 2020).

The genes involved in viral infection reported in the literature are *ACE2*, *TMPRSS2*, *ADAM17*, *NRP1*, and *NRP2*. One literature review (Fulzele et al., 2020) identified 12 relevant microRNAs in the regulation of *ACE2* (miR-18, miR-125b, miR-132, miR-143, miR-181, miR-200, miR-145, miR-155, miR-212, miR-421, miR-482-3p, and miR-4262). In addition to these microRNAs experimentally deduced to target *ACE2*, an *in silico* search was performed in the miRDB database (Chen and Wang, 2020) for possible microRNAs targeting the *ACE2*, *TMPRSS2*, *ADAM17*, *NRP1*, and *NRP2* genes. Only microRNAs with a score greater than 94 were considered. The search identified candidate microRNAs to be relevant biomarkers in the regulation of these genes. Additionally, seven microRNAs were described as candidates targeting the mRNAs of viral genes (Fulzele et al., 2020), making them an important group of markers.

Finally, a relevant group of microRNAs has been described as regulators of the inflammatory response (Tahamtan et al., 2018), of which some belong to the aforementioned list of microRNAs involved in regulating the expression of genes related to SARS-CoV-2 infection or targeting viral genes. Table 3 lists the microRNAs that may be relevant in COVID-19.

**TABLE 3:** MicroRNAs possibly relevant in COVID-19, according to their type of evidence.

***MicroRNAs with in silico evidence of regulation of the expression of genes related to SARS-CoV-2 infection:***
hsa-miR-124-3p, hsa-miR-1297, hsa-miR-153-5p, hsa-miR-26a-5p, hsa-miR-26b-5p, hsa-miR-3133, hsa-miR-3163, hsa-miR-331-3p, hsa-miR-33a-3p, hsa-miR-3646, hsa-miR-4465, hsa-miR-4500, hsa-miR-506-3p, hsa-miR-5094, hsa-miR-548ae-3p, hsa-miR-548ah-3p, hsa-miR-548aj-3p, hsa-miR-548am-3p, hsa-miR-548aq-3p, hsa-miR-548j-3p, hsa-miR-548x-3p, hsa-miR-578, hsa-miR-6844, hsa-miR-7977, hsa-miR-92a-1-5p
***MicroRNAs with experimental evidence of regulation of* ACE2 *expression:***
hsa-miR-125b, hsa-miR-145, hsa-miR-181, hsa-miR-200, hsa-miR-212, hsa-miR-421, hsa-miR-482-3p, hsa-miR-18, hsa-miR-132, hsa-miR-143, hsa-miR-155, hsa-miR-4262
***MicroRNAs with in silico evidence of regulation of viral gene expression:***
hsa-miR-15b-5p, hsa-miR-15a-5p, hsa-miR-548c-5p, hsa-miR-548d-3p, hsa-miR-409-3p, hsa-miR-30b-5p, hsa-miR-505-3p
***MicroRNAs with evidence of regulation of the inflammatory response*:**
hsa-miR-21, hsa-miR-24, hsa-miR-124, hsa-miR-145, hsa-miR-146, hsa-miR-149, hsa-miR-155, hsa-miR-181a, hsa-miR-181b, hsa-miR-181c, hsa-miR-181d

Correlating the most recent findings, host genetics linked to the immune response are strongly suggested as a predictor of the prognosis of COVID-19. Initial evidence pointing to genes such as *ACE2* as important for viral entry into the host cell is not as relevant as was expected. Rather, viral entry is now more clearly linked to genes such as *TMPRSS2* and *MX1*, which are near each other on chromosome 21. TMPRSS2 is crucial for the initial phenomena of infection and is responsible for the viral response related to interferon.

In this scenario, evidence emerged for specific immune response genes, such as KIR and MHC genes, in addition to a potential association with multiple PID genes. Therefore, the current data suggest that the influence of human host genetics on COVID-19 seems to be polygenic and focused on the genetic modulation of the immune response, making it increasingly less likely that the broad spectrum of COVID-19 manifestations is determined by oligogenic models. Additionally, the relevant role of multiple immunorelevant genes also favors the role of epigenetic regulation in these genes, especially microRNAs.

There are several pieces of evidence on the role of host genetics influencing the dynamics of SARS-CoV-2 infection, the variability of genes involved in the immune response has a direct impact on the course of COVID-19, but the magnitude of genetic diversity makes this elucidation more complex. However, concentrating efforts in order to discover the key genes involved in viral pathogenesis, as well as in the escape of the immune response and, based on this, building a panel with the main findings, can strongly contribute to the targeting of cases, enabling a more accurate view on how the evolution of cases can occur, and thus outline a more effective preventive and therapeutic planning, contributing to a better dynamics of care services, as well as favoring the studies of vaccines used today and those that are still under research.

## Transmission

The transmissibility of SARS-CoV-2 is not known with precision. It is believed that the ingestion of infected animals as a food source is the main cause of zoonotic transmission (Ji et al., 2020). As SARS-CoV-2 is highly similar to SARS-CoV, bats could be the host of the new coronavirus. In addition, Malaysian pangolins (*Manis javanica*) can harbor coronaviruses very similar to SARS-CoV-2 and are a potential natural reservoir of the virus (Zhou P. et al., 2020). Human-to-human transmission can occur through droplets containing infectious particles spread by speech, coughing, or sneezing, which can reach the mucous membranes of the eyes, nose, or mouth as portals, or by direct contact with contaminated surfaces, such as stainless steel, plastic, glass, and cardboard for at least several hours (Doremalen et al., 2020; Fan et al., 2020; Xiang Ong et al., 2020). As respiratory viruses have the highest transmission rate when the patient is symptomatic, because it is during this period that the viral load reaches a peak, it is believed that the same occurs with COVID-19 (Zhou F. et al., 2020). However, the possibility of viral transmission from an asymptomatic individual (Bai et al., 2020) is not excluded because there is evidence of asymptomatic or presymptomatic spread of SARS-CoV-2, highlighting its ability to colonize and replicate in the throat during the initial infection (Arons et al., 2020; Pan et al., 2020; Wölfel et al., 2020).

The genetic material of SARS-CoV-2 has been detected in the feces, whole blood, and urine of patients with COVID-19, but it has not been documented whether transmission by these means is possible (Young et al., 2020). The possibility of fecal aerosol transmission was described in a report based on circumstantial evidence, and this may have caused the community outbreak of COVID-19 in a high-rise building in Canton, China (Kang et al., 2020). Little is known about the vertical transmission of SARS-CoV-2, and further studies are needed to assess its transmissibility from pregnant woman to fetus (Chen H. et al., 2020; Hu X. et al., 2020). However, in March 2020, the first proven case of transplacental transmission of SARS-CoV-2 was described, involving a pregnant woman affected by COVID-19 during late pregnancy, with detection of the viral genome in the amniotic fluid collected before rupture of the placenta (Vivanti et al., 2020).

To date, there is no evidence of viral transmission from pets to humans. Ferrets and cats are highly susceptible to SARS-CoV-2, while dogs have low susceptibility; other animals, including pigs, chickens, and ducks, are not susceptible to the virus under experimental conditions (Shi et al., 2020). Interestingly, viral transmission between cats has been observed (Shi et al., 2020). Another study showed that 22 cats in France and two of 10 cats in China from patients with COVID-19 had SARS-CoV-2 infection with mild respiratory and digestive symptoms (Sailleau et al., 2020). This indicates that cats, being common pets, can theoretically transmit the virus to other animals and, possibly, humans. Still, there is no clear evidence that transmission of SARS-CoV-2 has occurred from cats to humans. In ferrets, SARS-CoV-2 is able to replicate in the upper respiratory tract without causing serious illness or death (Shi et al., 2020). Recently, an outbreak of SARS-CoV-2 in visions (Neovison mink) was reported on several farms in the Netherlands with transmission events to humans via respiratory droplets promoting secondary transmission of a mink SARS-CoV-2 variant back for humans (Oude Munnink et al., 2021; Shuai et al., 2021). Between April 26 and November 22, 2020, 14 outbreaks of COVID-19 occurred on commercial mink farms in Utah, one outbreak in a commercial mink farm in Wisconsin, and another in Oregon, USA (United States of America). Clinical signs included respiratory signs and sudden death from a total of 12,330 deaths among 145,757 susceptible animals (Eckstrand et al., 2021).

Repeat infections of SARS-CoV-2 between humans and animals (spillback) can lead to the emergence of new animal reservoirs, with risk of secondary infection (spillover) for humans through an animal reservoir, which can lead to the appearance of variants of SARS-CoV-2, as described above. Such events are of great concern, as the formation of wild virus reservoirs, the appearance of a mutant strain with increased transmissibility and severity of SARS-CoV-2 in humans puts efforts for the long-term control of COVID-19 at risk and they also threaten vulnerable animal populations that are particularly susceptible to lethal diseases.

## Diagnosis of COVID-19

Faced with the pandemic caused by the new coronavirus, the early and safe diagnosis of the infection is extremely important to interrupt the transmission of the disease and assist in making decisions such as isolation and/or distancing of people, which will provide more time for public health implementation measures that may have positive impacts in reducing the problems associated with COVID-19.

According to the WHO, the diagnosis of COVID-19 can be clinical or epidemiological, using the International Code of Diseases (ICD) ICD-10 Z20.9 (contact with exposure to unspecified communicable disease) as a record. In the face of clinical manifestations suggestive of the disease and when laboratory confirmation is inconclusive or not available, the patient is considered infected and the ICD UO7.2 (unidentified virus - attributed to a clinical or epidemiological diagnosis of COVID) should be used for recording –19, when laboratory confirmation is inconclusive or unavailable. Includes diagnosis of a probable case or suspected case of COVID-19). On the other hand, those with a diagnosis confirmed by laboratory tests must be registered using the code ICD-UO7.1 (identified virus – attributed to a COVID-19 diagnosis confirmed by laboratory tests) (Brazil Ministério da Saúde, 2020).

**FIGURE 2:**
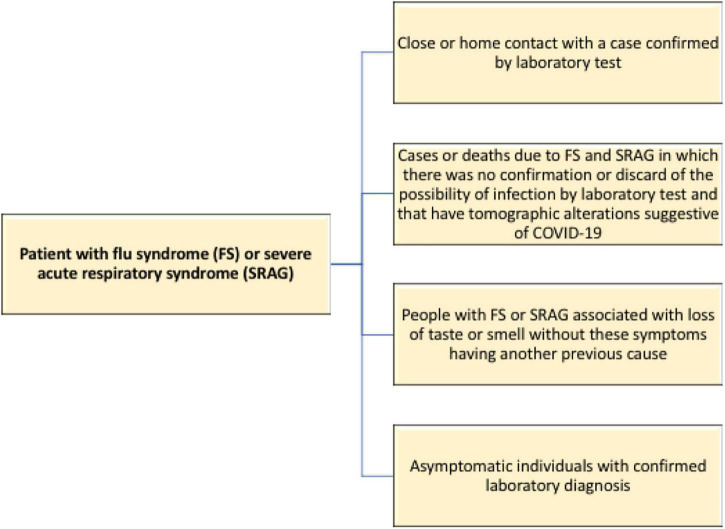
Flowchart for identifying individuals possibly infected with the new coronavirus in the absence of laboratory tests.

The Brazilian Ministry of Health included new criteria for the characterization of COVID-19 cases, going beyond the laboratory tests already adopted. Given the difficulties of testing, the agency allowed for the following individuals to be deemed infected by the new coronavirus (Brazil Ministério da Saúde, 2020; Figure 2).

SARS-CoV-2 was initially characterized by sequencing its genome, which provided the necessary information to develop quantitative or real-time polymerase chain reaction (PCR) tests for viral detection (Zhou P. et al., 2020; Zhu et al., 2020). Different laboratory diagnostic tools are used in clinical practice to confirm cases of SARS-CoV-2 infection and differ in terms of sample type, collection time, specificity, and sensitivity (Table 4).

**TABLE 4:** Diagnostic methods for SARS-CoV-2 infection.

Test	Type of sample	Pros and cons	References
RT-PCR	Nasopharyngeal smear or saliva. Collection within 7 days of symptoms	Gold standard test for detecting SARS-CoV-2. The accuracy of the test depends on the stage of the disease and the degree of viral multiplication. Higher sensitivities are reported depending on which genetic targets are used in performing the test	Xiao et al., 2020; Zou et al., 2020; Erster et al., 2021
Enzyme-Linked Immunosorbent Assay (ELISA) IgM, IgA, IgG	Serum	Useful for diagnosing previous infections. Important for population serological surveillance and research activities. It is not useful for diagnosing acute infection. Descending titles over time (2–3 months)	Huang A. T. et al., 2020; Huang M. et al., 2020; Krajewski et al., 2020; Li et al., 2020; Lucas et al., 2020; Bichara et al., 2021
Lateral flow immunoassay (LFIA) (Antigen or antibody)	Nasal or nazopharinzeal smear, serum or whole blood	Detects acute infection through the presence of viral antigens. Previous infection by detection of antibodies. Average time 15–20 min. Low cost. Low sensitivity and specificity of 56.2 and 99.5%, respectively	Canetti et al., 2020; Dinnes et al., 2020; Prazuck et al., 2020
Loop-mediated isothermal amplification (LAMP)	Nasal or nazopharinzeal smear	The accuracy of the test depends on the stage of the disease and the degree of viral multiplication. Highly effective, fast results, and limited cost	Broughton et al., 2020; Huang W. E. et al., 2020; Bektaş et al., 2021; Jones et al., 2021; Juscamayta-López et al., 2021
CRISPR/Cas	Nasal or nazopharinzeal smear	High sensitivity and specificity rates and low analysis costs. With 100% sensitivity and specificity	Broughton et al., 2020; Hou et al., 2020; Lucia et al., 2020
Biosensors	Nazopharinzeal smear	Tecnologia rápida e altamente sensível. Ausência de reatividade cruzada com outros coronavirus	Seo et al., 2020; Zhao et al., 2021

The COVID-19 pandemic highlighted the importance of laboratory diagnostic methods. Currently, nucleic acid amplification methods represent the gold standard for the diagnosis of COVID-19 infection with several RT-PCR-based tests approved by different national and international regulatory agencies. However, despite the high sensitivity of RT-PCR, the need for trained professionals and expensive instruments and reagents that limit its application, especially in low-income countries, drive the development of diagnostic methods to overcome the limitations of RT-PCR, among them, low-cost diagnostic strategies are promising and can be used for the effective diagnosis of COVID-19 infection in low- and middle-income countries. Rapid antigen and antibody tests and immunoenzymatic serological tests represent the most widely used techniques for monitoring the spread of SARS-CoV-2 infection. It is important to note that, despite the low cost of such techniques, the low sensitivity and specificity of LFIAs and ELISAs, the use of such point-of-care tests enabled the implementation of effective health surveillance systems that allowed for the effective management of the COVID pandemic -19, thus limiting the number of infections.

The scientific community in a short period developed several useful methods to correctly diagnose a suspected case of SARS-CoV-2 infection. Despite the limitations of some laboratory diagnostic methods, it is necessary to take into account not only the test to be used but also the patient’s medical history, the time of exposure to individuals infected with SARS-CoV-2, the type of sample to be used, be collected and analyzed, and how to interpret the result. The integration of all these elements will provide a solid foundation for correctly diagnosing COVID-19 infection and effectively managing the COVID-19 pandemic.

## Treatment Approaches

According to the WHO, there are still no specific drugs for COVID-19. In the early days of the pandemic, many governments around the world implemented, to some degree, measures recommended by the WHO to limit the spread of the virus, such as self-isolation, social distancing, hand washing, closing schools and universities, and mask wearing in public places (Prather et al., 2020). Due to the lack of specific therapy, some drugs used in other infectious diseases have been applied against COVID-19 (Table 5) in clinical practice, although their effectiveness is controversial.

**TABLE 5:** Medicines used in the treatment of COVID-19.

Drug name	Class	Mechanism of action	Adverse effect	References
Chloroquine and Hydroxychloroquine	Antiparasitic	Inhibition of host cell receptor glycosylation to block viral entry, acidification of the endosomal and proteolytic process.	High doses can lead to respiratory arrest, cardiac arrest, and hypokalemia.	Savarino et al., 2003; Yamamoto et al., 2004; Eze et al., 2021
Lopinavir/Ritonavir	Antiviral	Inhibition of 3CL protease. Inhibition of viral replication.	Risks for pediatric patients	Chu et al., 2004; Kim et al., 2016
Remdesivir	Antiviral	RNA-dependent RNA polymerase inhibitor. Block viral replication	There is no information on whether overdosing can cause any adverse effects	Hoehl et al., 2020; Li and De Clercq, 2020; Wang et al., 2020e
Heparin	Anticoagulant and anti-inflammatory	Heparin binds to the RBD of the SARS-CoV-2 protein S, inhibiting viral infection	Platelet count usually decreases to between days 5 and 12	Kawase et al., 2012; Zhou et al., 2015; Hoffmann et al., 2020; Mycroft-West et al., 2020; Tang N. et al., 2020; Yamamoto et al., 2020
Tocilizumab	Monoclonal antibody	IL-6 inhibiting receptor. Cytokine storm reduction blockade.	Overdose-neutropenia	Guaraldi et al., 2020; Rosas et al., 2020
Anakinra	Immune Response Modulator	Monoclonal antibody that acts against the IL-1 receptor	Rheumatoid arthritis (incidence > 10%)	Conti et al., 2020
Baricitinib	Immune Response Modulator	Antiviral activity Inhibitor of clathrin-mediated endocytosis Janus kinases 1 and 2 (JAK1/2 inhibitor)	Multiple adverse reactions	Cantini et al., 2020; Richardson et al., 2020
Camostat Mesilate	Antiviral	TMPRSS2 inhibitor that prevents replication Viral Blocks viral mutation	Rash, pruritus, nausea, abnormal values from laboratory tests and diarrhea	Uno, 2020
Molnupiravir	Antiviral	It works by inducing mutagenesis in viral RNA, causing the newly formed RNA strand chain to terminate	Mild adverse effects	Kabinger et al., 2021; Zhou et al., 2021
Paxlovid	Antiviral	It inhibits viral replication at a stage known as proteolysis, which occurs before viral RNA replication	Absent	Wang et al., 2020g; Ahmad et al., 2021; Pfizer, 2021 https://www.pfizer.com/news/press-release/press-release-detail/pfizers-novel-covid-19-oral-antiviral-treatment-candidate

The unfortunate SARS-CoV-2 pandemic in early 2020 posed a challenge to all researchers to find potential therapeutic agents for treatment. There were extensive efforts to reuse approved drugs during the COVID-19 pandemic. This strategy offers several advantages over developing an entirely new drug, with reduced risk of failure because safety has already been evaluated. Currently, there are antiviral therapies developed to induce a direct effect on SARS-CoV-2, either by blocking viral entry into host cells or by controlling viral enzymes with a significant contribution to genome replication. A big step forward in the control of severe cases and deaths related to COVID-19.

## Vaccine Platforms

Multiple vaccine platforms have already been approved for emergency use against COVID-19. As of August 19, 2021, 20 vaccine candidates were within the WHO evaluation process for commercialization (World Health Organization [WHO], 2021b; Table 6).

**TABLE 6:** Vaccines available and in development for protecting against SARS-CoV-2.

Manufacturer	Vaccine name	Platform	Evaluation status	Status
BioNTech Manufacturing GmbH	BNT162b2/COMIRNATY Tozinameran (INN)	Nucleoside modified mRNA	Finished	Approved for use
AstraZeneca, AB	AZD1222 Vaxzevria	Recombinant ChAdOx1 adenoviral vector encoding the Spike protein antigen of the SARS-CoV-2.	Finished	Approved for use
Serum Institute of India Pvt. Ltd	Covishield (ChAdOx1_nCoV-19)	Recombinant ChAdOx1 adenoviral vector encoding the spike protein antigen of the SARS-CoV-2.	Main data finalized	Approved for use
Janssen–Cilag International NV	Recombinant, Ad26.COV2.S	Recombinant, replication-incompetent adenovirus type 26 (Ad26) vectored vaccine encoding the (SARS-CoV-2) spike protein	Finished	Approved for use
Moderna Biotech	mRNA-1273	mRNA-based vaccine encapsulated in lipid nanoparticle (LNP)	Finished	Approved for use
Beijing Institute of Biological Products Co., Ltd. (BIBP)	SARS-CoV-2 Vaccine (Vero Cell), Inactivated (lnCoV)	Inactivated, produced in Vero cells	Finished	Approved for use
Sinovac Life Sciences Co., Ltd.	COVID-19 Vaccine (Vero Cell), Inactivated/CoronavacTM	Inactivated, produced in Vero cells	Finished	Approved for use
The Gamaleya National Center	Sputnik V	Human Adenovirus Vector-based COVID-19 vaccine	Waiting, waiting for submission	Approved for use
Bharat Biotech, India	SARS-CoV-2 Vaccine, Inactivated (Vero Cell)/COVAXIN	Whole-Virion Inactivated Vero cells	In progress	Approved for use
Sinopharm/WIBP	Inactivated SARS-CoV-2 Vaccine (Vero Cell)	Inactivated, produced in Vero cells	In progress	In progress. Not approved
CanSinoBio	Ad5-nCoV	Recombinant novel coronavirus vaccine (adenovirus type 5 vector)	-	In progress. Not approved
Nonavax	NVX-CoV2373/Covovax	Recombinant nanoparticle prefusion spike protein formulated with Matrix-M™ adjuvant	–	In progress. Not approved
Sanofi	CoV2 preS dTM-AS03 vaccine	Recombinant, adjuvanted	–	In progress. Not approved
Serum Institute of India Pvt. Ltd	NVX-CoV2373/Covovax	Recombinant nanoparticle prefusion spike protein formulated with Matrix-M™ adjuvant	–	In progress. Not approved.
Clover Biopharmaceuticals	SCB-2019	Novel recombinant SARS-CoV-2 spike (S)-Trimer fusion protein	–	In progress. Not approved
Urevac	Zorecimeran (INN) concentrate and solvent for dispersion for injection; Company code: CVnCoV/CV07050101	mRNA-based vaccine encapsulated in lipid nanoparticle (LNP)	–	In progress. Not approved
Vector State Research Center of Virology and Biotechnology	EpiVacCorona	Peptide antigen	–	In progress. Not approved
Zhifei Longcom	Recombinant Novel Coronavirus Vaccine (CHO Cell)	Recombinant protein subunit	–	In progress. Not approved
IMBCAMS	SARS-CoV-2 Vaccine, Inactivated (Vero Cell)	Inactivated	–	In progress. Not approved
BioCubaFarma	Soberana 01, Soberana 02 Soberana Plus Abdala	SARS-CoV-2 spike protein conjugated chemically to meningococcal B or tetanus toxoid or aluminum	–	In progress. Not approved

*Adapted from World Health Organization [WHO] (2021c). –, No information.*

The efficacy of an effective vaccine depends on the long-term response of specific antibodies to viral antigens from plasma cells, as well as the development of persistent memory of T cells and B cells. In the case of SARS-CoV infection, adaptive humoral and cellular immune responses are crucial to the elimination of infection.

Due to the need to ensure the immunity of the population, clinical trials of SARS-CoV-2 vaccines have accelerated their development process. To date, few adverse events have been reported in clinical trials of such vaccines, and the safety of those that have been tested more extensively is promising.

Along with safety, the efficacy of the vaccine is important. The FDA recommended that vaccines show at least 50% efficacy compared to placebo, defined primarily by (i) reduction in COVID-19 cases, (ii) reduction in COVID-19 severity, or (iii) reduction in COVID-19 severity infections by SARS-CoV-2 (Food and Drug Administration [FDA], 2020).

### Inactivated Virus

Inactivated virus vaccines are being developed by isolating SARS-CoV-2 from samples of patients hospitalized with COVID-19. Then the virus is used to infect a cell line in the laboratory, after which it is chemically inactivated with β-propiolactone (Gao et al., 2020). This type of platform exposes the vaccinated individual to several viral proteins instead of a single target, and depending on the inactivation process, the structural integrity of the viral antigens can be affected to favor a T cell response toward a Th2 profile (Tregoning et al., 2020). In 2020, a pilot study of a candidate inactivated SARS-CoV-2 vaccine (BBIBP-CorV) demonstrated high productivity and good genetic stability over the SARS-CoV-2 vaccine (Wang et al., 2020h).

### Attenuated Virus

This type of platform simulates natural infection using deoptimized viral genomes that are not translated efficiently in hosts (Tregoning et al., 2020). With a low level of infection, these vaccines induce robust and long-lasting immune responses (Yong et al., 2019). However, the storage of these vaccines at low temperature and their contraindication in immunocompromised patients and the elderly may be considered disadvantages (Sarmiento et al., 2016).

### Viral Vectors

Vaccines using recombinant virus vectors work similarly to an endogenous pathogen, expressing the target protein in the cytoplasm of the host cell. This location favors the response of cytotoxic T cells, establishing the cell-mediated immunity that is crucial for the elimination of virus-infected cells (Coughlan, 2020). Vaccines using adenoviral vectors can induce potent antibodies and T cell responses, with variations in intensity depending on the serotype (Tan et al., 2004). Currently, there are two vaccine candidates against SARS-CoV-2 in phase I/II clinical trials that use the measles virus (NCT04497298) or the vesicular stomatitis virus (NCT04569786) as a vector (Jon, 2020).

### Nucleic Acid

In addition, DNA or RNA vaccination is also able to trigger humoral and cellular immune responses through the activation of CD4^+^ helper T cells and CD8^+^ cytotoxic T cells, respectively. Upon entering the cell, DNA vaccines are detected by a variety of innate immune receptors, such as interferon gene stimulator (STING)/TANK binding kinase 1 (TBK1). The IRF3 pathway, the inflammasome, and many other factors are involved in the mode of action of the DNA vaccine (Li and Petrovsky, 2016). Two studies have analyzed the immunogenicity of these vaccine platforms against SARS-CoV-2 in animal models (Smith et al., 2010; Patel et al., 2020).

### Recombinant Protein Subunit

Subunit vaccines, particularly those based on the RBD of the SARS-CoV S protein, contain the main antigenic determinants that can induce neutralizing antibodies and CD8^+^ T cell responses (Bonavia et al., 2003; He et al., 2006). This characteristic provides useful information for designing safe and effective vaccines against SARS-CoV-2, since the RBD of the S protein of SARS-CoV-2 contains similar epitopes. However, they are vaccines that generally require adjuvants or nanoparticles to increase their immunogenicity. A subunit vaccine (StriFK-FH002C) prevented hamsters from transmitting the virus to other, unvaccinated hamsters in cohabitation, causing a lower viral load in the upper respiratory tract of hamsters vaccinated after the challenge, showing that the vaccine is a candidate for SARS-CoV-2 (Wu et al., 2021).

Despite this success, the goal of achieving global herd immunity through vaccination, which would allow for the abandonment of other non-pharmaceutical interventions and restrictions on our social, cultural, and recreational activities has not yet been achieved in all countries. This is due to a number of reasons and unexpected developments that challenged and delayed the progress of vaccination. One of the most striking developments has been the rapid local and global emergence of SARS-CoV-2 variants with different transmission and immune evasion properties. This not only started new waves of infections but also impacted the evaluation of the vaccine’s efficacy in clinical trials. Another major obstacle to global immunity is represented by a strong imbalance in the worldwide distribution of vaccines. A global pandemic strategy combined between prevention measures and vaccination will minimize the burden of infection for the poor, but also for rich countries, and depends on the equitable distribution of vaccines to establish large-scale immunity at the global level and contain the COVID-19 pandemic.

## Socioeconomic Aspects of the Pandemic

Individuals affected by COVID-19 are potentially at risk of physiological and economic harm. The decline of the economy began with the decrease in activity in the travel, tourism, and export sectors (Organization for Economic Cooperation and Development [OECD], 2020; Khan et al., 2020), but soon its generalized effects hindered production and consumption, with layoffs and bankruptcies in all sectors.

In 2020, the pandemic scenario accentuated the decline in the economies of Latin American and Caribbean countries, which have low economic growth (Organization for Economic Cooperation and Development [OECD], 2020). This scenario, together with the economic slowdown faced in previous years and the drop in activity caused by the pandemic, has negatively affected living standards and well-being in the countries of the region (Organization for Economic Cooperation and Development [OECD], 2020). The most vulnerable population will be low-income people, approximately 74% of whom work informally (Organization for Economic Cooperation and Development [OECD], 2020). By itself, the pandemic attempts to affect the present and the future with regard to the aspect of life of the people, which includes material goods (income, quality of employment, and housing) and aspects such as education, ability to form skills, in addition to emotional well-being (Khan et al., 2020; Organization for Economic Cooperation and Development [OECD], 2020).

The global economic recovery is predicted to be slow; for 2021, it will increase almost 6% differently from the previous year, with an increase of 3.5%; however, this is far from sufficient to ensure the necessary impetus for growth of the gross domestic product worldwide (Organization for Economic Cooperation and Development [OECD], 2021). With the advancement of large-scale vaccination, the manufacturing sector is gradually growing as trade and the reopening of borders progress, promoting an increase in job creation (Organization for Economic Cooperation and Development [OECD], 2021). In contrast, there is caution about the arrival of enough vaccines for enterprises of low-income individuals, especially in underdeveloped or developing countries, enabling a further weakening of economic growth, with acute increase in poverty and potentially in financing problems because the global economic and social impact of keeping borders closed outweighs the costs of making vaccines, tests, and health supplies more widely available for these countries (Khan et al., 2020; Organization for Economic Cooperation and Development [OECD], 2021).

With the increase in the unemployment rate, governments and central banks should intervene with increased spending and lower interest rates to increase consumer demand and investment, respectively (International Labour Organization [ILO], 2020). Even so, estimating the economic costs of a global disease at this time is still uncertain, since the pandemic has spiraling effects on the national and global economy, which means that any economic shock in a country will quickly spread to other countries due to the commercial and financial links associated with globalization (International Labour Organization [ILO], 2020).

Low- and middle-income countries remain vulnerable to the pandemic, in addition to suffering dramatic social and economic consequences. In this scenario, ensuring the emergence and success of the adoption of new forms of economic development and governance models would not only help to reduce the socioeconomic discrepancies affected by the pandemic, but also the risk associated with vulnerable populations. These social changes must be the result of a reflection that enables the generation of new behaviors, and reflections and actions on the socioeconomic aspects of the pandemic. However, this issue raised by COVID-19’s control policies appears to have received little attention in the relevant economic literature. And with that, the outbreak aggravated existing vulnerabilities, injustices, and distrust in society.

## Final Considerations

This study presents an overview of the current context of COVID-19, offering a summary of the effects on health and socioeconomic status, viral characteristics, transmission, therapeutic options, vaccine prospects, immune response, and available diagnostic tools. It is possible that only when the pandemic ends will we be able to more accurately assess the economic and social consequences of this catastrophic event and that only then will we be able to extract sufficient knowledge to fight future epidemics, especially in matters of global public health.

## Author Contributions

AV conceptualized the idea of the article for review. MT performed the literature search, analyzed the cited articles, and wrote the manuscript. CB, MV, MQ, IV, ES, CC, and MA carefully reviewed the manuscript and made changes and additions to its intellectual content. All authors approved the submitted version.

## Conflict of Interest

The authors declare that the research was conducted in the absence of any commercial or financial relationships that could be construed as a potential conflict of interest.

## Publisher’s Note

All claims expressed in this article are solely those of the authors and do not necessarily represent those of their affiliated organizations, or those of the publisher, the editors and the reviewers. Any product that may be evaluated in this article, or claim that may be made by its manufacturer, is not guaranteed or endorsed by the publisher.

## Footnotes

1
https://www.orpha.net/consor/cgi-bin/index.php
