# Supplementary material for: The Complexity of SARS-CoV-2 Infection and the COVID-19 Pandemic
Source: Front Microbiol. 2022 Feb 10;13:789882. doi: 10.3389/fmicb.2022.789882 (PMC8870622; doi:10.3389/fmicb.2022.789882)
Supplement: Supplementary file 1 [file Table_1.DOCX]

Supplementary Material

Supplementary file. Comprehensive view of the cytogenetic regions with evidences of association with COVID 19 severity and their respectives types of study, including genomic surveys, candidate genes case control studies, metaanalysis, functional studies and presence of Primary immunodeficiency genes in the regions. Additionally, candidate genes and their respective references are also presented.

| **Region** | **Score** | **GWAS** | **WES** | **WGS** | **CCS** | **MAS** | **FS** | **PIDG** | **Candidate genes** | **References** |
| --- | --- | --- | --- | --- | --- | --- | --- | --- | --- | --- |
| 10p15.2 | 1 |  |  |  |  |  | 1 |  | *KLF6* | (Shaath et al., 2020) |
| 10q26.13 | 1 |  |  | 1 |  |  |  |  | *HMX2* | (Wang et al., 2020d) |
| 11p15.5 | 3 |  | 1 |  |  |  | 1 | 1 | *IRF7, IFITM1, IFITM2* | (Shaath et al., 2020) |
| 11q12.2 | 2 |  |  |  |  | 1 |  | 1 | *CD20* | (Rendeiro et al., 2021) |
| 11q13.1 | 2 |  |  |  |  |  | 1 | 1 | *MALAT1, NEAT1* | (Shaath et al., 2020) |
| 11q13.2 | 2 |  | 1 |  |  |  |  | 1 | *UNC93B1* | (Zhang et al., 2020c) |
| 12p13.2 | 2 |  |  |  |  |  | 1 | 1 | *KLRC2, NKG2C* | (Maucourant et al., 2020; Vietzen et al., 2020) |
| 12q14.2 | 2 |  |  |  |  |  |  | 1 | *TBK1* | (Zhang et al., 2020c) |
| 12q24.13 | 2 | 1 |  |  |  |  |  | 1 | *OAS1-OAS3* | (Pairo-Castineira et al., 2021) |
| 12q24.33 | 2 |  |  | 1 |  |  |  | 1 | *GOLGA3* | (Wang et al., 2020d) |
| 15q.26.1 | 2 |  |  |  |  |  | 1 | 1 | *FURIN* | (Secolin et al., 2021) |
| 16p11.2 | 2 |  |  |  |  | 1 |  | 1 | *CD19* | (Rendeiro et al., 2020) |
| 16p13.3 | 2 |  |  |  |  |  | 1 | 1 | *CLUAP1* | (Hu et al., 2020a) |
| 17q11.1 | 1 |  |  |  |  |  | 1 |  | *WSB1* | (Hu et al., 2020a) |
| 17q12 | 2 |  |  |  |  | 1 | 1 |  | *CCL8, CCL3, CCL2, CCL5* | (Shaath et al., 2020; Vastrad et al., 2020) |
| 17q25.1 | 1 |  |  |  |  |  | 1 |  | *H3F3B* | (Shaath et al., 2020) |
| 19p13.11 | 2 |  |  |  |  |  | 1 | 1 | *HLA-DPA1* | (Birol, 2020) |
| 19p13.2 | 3 | 1 |  |  | 1 |  |  | 1 | *TYK2, CLEC4M (L-SIGN), DC-SIGN* | (Amraei et al., 2020; Pairo-Castineira et al., 2021) |
| 19p13.3 | 3 | 1 | 1 |  |  |  |  | 1 | *C3,CFD, ELANE, TICAM1, DPP9* | (Ellinghaus et al., 2020; Zhang et al., 2020a; Pairo-Castineira et al., 2021) |
| 19q13.32 | 2 |  |  |  |  |  | 1 | 1 | *APOC1, APOE* | (Shaath et al., 2020) |
| 19q13.33 | 2 |  | 1 |  |  |  |  | 1 | *IRF3* | (Zhang et al., 2020c) |
| 19q13.42 | 4 |  |  |  | 1 | 1 | 1 | 1 | *KIR gene Cluster* | (Rendeiro et al., 2020; Sakuraba et al., 2020; Bernal et al., 2021) |
| 1p36.12 | 2 |  |  |  |  |  | 1 | 1 | *C1QB* | (Shaath et al., 2020) |
| 1q21.3 | 2 |  |  |  |  |  | 1 | 1 | *S100A8* | (Shaath et al., 2020) |
| 1q32.2 | 2 |  |  |  |  | 1 |  | 1 | *CD55* | (Vastrad et al., 2020) |
| 20q13.13 | 1 |  |  | 1 |  |  |  |  | *TMEM189-UBE2V1* | (Wang et al., 2020d) |
| 21q22.11 | 4 | 1 | 1 |  |  | 1 |  | 1 | *IFNAR1, IFNAR2* | (Vastrad et al., 2020; Zhang et al., 2020a; Pairo-Castineira et al., 2021) |
| 21q22.3 | 6 |  | 1 | 1 | 1 | 1 | 1 | 1 | *TPRSS2, MX1* | (Andolfo et al., 2020; Asselta et al., 2020; Vastrad et al., 2020; Wang et al., 2020d; Bizzotto et al., 2020; Latini et al., 2020; Mohammad et al., 2020; Russo et al., 2020; Sajuthi et al., 2020; Sanchis P et al., 2020; Senapati et al., 2020; Torre-Fuentes et al., 2021; Schönfelder et al., 2021) |
| 22q11.21 | 2 |  |  |  |  | 1 |  | 1 | *BID* | (Vastrad et al., 2020) |
| 2p25.1 | 2 |  |  |  |  |  | 1 | 1 | *ADAM17* | (Lambert et al., 2005; Heurich et al., 2014; Gemmati et al., 2020) |
| 2q32.2 | 2 |  |  |  |  | 1 |  | 1 | *STAT1* | (Vastrad et al., 2020) |
| 2q32.3 | 1 |  |  |  |  |  | 1 |  | *DNAH7* | (Hu et al., 2020a) |
| 2q35 | 2 |  |  |  |  |  | 1 | 1 | *DES, SPEG* | (Hu et al., 2020a) |
| 3p21.31 | 3 | 1 |  |  |  |  | 1 | 1 | *TREX1, LZTFL1, SLC6A20, FYCO1, CXCR6, XCR1, CCR9* | (Pairo-Castineira et al., 2021; Secolin et al., 2021) |
| 3q13.33 | 1 |  |  |  |  | 1 |  |  | *CD80* | (Vastrad et al., 2020) |
| 4q12 | 2 |  |  | 1 |  |  |  | 1 | *NOA1* | (Wang et al., 2020d) |
| 4q22.1 | 1 |  |  |  |  |  | 1 |  | *SPP1* | (Shaath et al., 2020) |
| 4q35.1 | 2 |  | 1 |  |  |  |  | 1 | *TLR3* | (Zhang et al., 2020a) |
| 5q31.1 | 2 |  |  |  |  |  | 1 | 1 | *IL13* | (Sajuthi et al., 2020) |
| 6p21.32 | 5 | 1 |  |  | 1 | 1 | 1 | 1 | *MHC Class II, NOTCH4, DQB1, HLA-DMA* | (Poulton et al., 2020; Spinetti et al., 2020; Vastrad et al., 2020; Wang et al., 2020c; Barquera et al., 2020; Benlyamani et al., 2020; Birol, 2020; Littera et al., 2020; Novelli et al., 2020; Amoroso et al., 2021; Anzurez et al., 2021; Romero-López et al., 2021; Schindler et al., 2021; Secolin et al., 2021; Langton et al., 2021; Naemi Fatmah M., Al-adwani Shurooq, Al-khatabi Heba, 2021; Pairo-Castineira et al., 2021) |
| 6p21.33 | 6 | 1 |  | 1 | 1 | 1 | 1 | 1 | *MHC class I, CCHCR1, HLA-B* | (Littera et al., 2020; Barquera et al., 2020; Sakuraba et al., 2020; Vastrad et al., 2020; Wang et al., 2020d, 2020c, 2020d; Birol, 2020; Novelli et al., 2020; Pairo-Castineira et al., 2021; Poulton et al., 2020; Bernal et al., 2021; Rosenbaum et al., 2021; Yung et al., 2021; Bonaccorsi et al., 2021; Khor et al., 2021; Leite et al., 2021) |
| 6p22.1 | 5 | 1 |  | 1 | 1 | 1 | 1 |  | *MHC class I, HLA-G, HLA-E* | (Barquera et al., 2020; Tomita et al., 2020; Toyoshima et al., 2020; Vietzen et al., 2020; Wang et al., 2020d; Ishii, 2020; Iturrieta-Zuazo et al., 2020; Littera et al., 2020; Poulton et al., 2020; Sakuraba et al., 2020; Correale et al., 2021; Schindler et al., 2021; Shkurnikov et al., 2021; Takagi e Matsui, 2021; Khor et al., 2021; Lorent et al., 2021; Pairo-Castineira et al., 2021) |
| 6q24.3 | 2 |  |  |  |  |  | 1 | 1 | *STXBP5* | (Hu et al., 2020a) |
| 7p15.3 | 1 |  |  |  |  |  | 1 |  | *IL-6, TOMM7* | (Hu et al., 2020a; Sajuthi et al., 2020) |
| 8q21.13 | 2 |  |  |  |  |  | 1 | 1 | *FABP4* | (Shaath et al., 2020) |
| 9p24.1+M48 | 1 |  |  |  |  | 1 |  |  | *JAK2* | (Vastrad et al., 2020) |
| 9q34.2 | 3 | 1 |  |  | 1 |  | 1 |  | *ABO* | (Ellinghaus et al., 2020; Jiao Zhao, Yan Yang, Hanping Huang, Dong Li, Dongfeng Gu, Xiangfeng Lu, Zheng Zhang, Lei Liu, Ting Liu, Yukun Liu, Yunjiao He, Bin Sun, 2020; Steven Lehrer, 2020; Wang et al., 2020c; Wu et al., 2020b; Wool e Miller, 2021) |
| 9q34.3 | 2 |  |  | 1 |  |  |  | 1 | *DPP7* | (Wang et al., 2020d) |
| Xp22.11 | 2 |  |  |  |  |  | 1 | 1 | *SAT1* | (Shaath et al., 2020) |
| Xp22.2 | 4 |  |  |  | 1 | 1 | 1 | 1 | *ACE2* | (Benetti et al., 2020; Bosso et al., 2020; Sajuthi et al., 2020; Strafella et al., 2020; Wu et al., 2020b; Cao et al., 2020; Gómez et al., 2020; Hubacek et al., 2020; Novelli et al., 2020; Torre-Fuentes et al., 2021; Calcagnile et al., 2021; Edelstein et al., 2021; Martínez-Sanz et al., 2021; Möhlendick et al., 2021) |

**LEGENDA: Region:** Cytogenetic region**; GWAS:** Genome Wide Association Studies; **WES**: Wide Exome Study; **WGS**:Wide Genome Study; **CCS**: Case Control Studies; **MAS:** Meta analysis Studies**; FS:** Funtional Studies; **PIDG**: Primary Immunodeficience Disease Genes.

**Score 4-6:** Strong Evidence.
